# Prediction of dinucleotide-specific RNA-binding sites in proteins

**DOI:** 10.1186/1471-2105-12-S13-S5

**Published:** 2011-11-30

**Authors:** Michael Fernandez, Yutaro Kumagai, Daron M Standley, Akinori Sarai, Kenji Mizuguchi, Shandar Ahmad

**Affiliations:** 1 Kyushu Institute of Technology, Fukuoka, Japan; 2 Immunology Frontier Research Center (IFReC), Osaka University, Japan; 3 National Institute of Biomedical Innovation, Japan

## Abstract

**Background:**

Regulation of gene expression, protein synthesis, replication and assembly of many viruses involve RNA–protein interactions. Although some successful computational tools have been reported to recognize RNA binding sites in proteins, the problem of specificity remains poorly investigated. After the nucleotide base composition, the dinucleotide is the smallest unit of RNA sequence information and many RNA-binding proteins simply bind to regions enriched in one dinucleotide. Interaction preferences of protein subsequences and dinucleotides can be inferred from protein-RNA complex structures, enabling a training-based prediction approach.

**Results:**

We analyzed basic statistics of amino acid-dinucleotide contacts in protein-RNA complexes and found their pairing preferences could be identified. Using a standard approach to represent protein subsequences by their evolutionary profile, we trained neural networks to predict multiclass target vectors corresponding to 16 possible contacting dinucleotide subsequences. In the cross-validation experiments, the accuracies of the optimum network, measured as areas under the curve (AUC) of the receiver operating characteristic (ROC) graphs, were in the range of 65-80%.

**Conclusions:**

Dinucleotide-specific contact predictions have also been extended to the prediction of interacting protein and RNA fragment pairs, which shows the applicability of this method to predict targets of RNA-binding proteins. A web server predicting the 16-dimensional contact probability matrix directly from a user-defined protein sequence was implemented and made available at: http://tardis.nibio.go.jp/netasa/srcpred.

## Background

Protein-RNA interactions are involved in various regulatory and constitutional cellular functions. Role for RNA in cellular defense and developmental regulation, which involves its interaction with proteins, has also been reported [1,2]. Most of these biological functions involve an accurate identification of specific recognition sites in proteins and RNA. Yet, protein-RNA interactions are far less understood than protein–DNA and protein-protein interactions, partly because fewer structures are available and also likely because these interactions are more complex than the others. In contrast to the intuitively similar protein-DNA interactions, nucleic acid structures in protein-RNA complexes are diverse, resulting in a wider range of mechanisms for protein-RNA interactions [3,4] and hence their prediction is more difficult.

Several computational approaches have been developed to identify RNA-binding proteins and RNA-binding sites. Statistical potentials and docking scoring functions derived from databases of complex crystal structures effectively identified RNA-binding sites in proteins [5-7]. Prediction of RNA-protein interactions from sequences or structures using machine learning methods have also been successfully attempted [8-15]. However, none of these methods addresses the crucial issue of specific interactions i.e. the same protein interacts with some RNA sequences and not others. Knowledge of exact interaction partners and the mechanism of recognition are essential for an understanding and ability to control RNA-level regulatory processes. Hence, an extension of machine learning methods to address this problem is of crucial significance.

We recently addressed the issue of specific recognition of protein and nucleotide subsequences for protein-DNA interactions by looking at the dinucleotide specific contact preferences of amino acids in proteins, using sequence and evolutionary contexts, and applied this information to predict specific contacts from sequence [16]. As a natural extension of this approach to RNA-binding proteins, and also because some RNA-binding proteins are shown to bind regions largely characterized by enrichment in one dinucleotide [17], we explored the possibility of predicting amino acid dinucleotide contacts. We start with a statistical analysis of protein-RNA contacts and identify patterns of contact preferences that contrast or resemble protein-DNA contacts. The prediction model takes a matrix of sequence- and evolutionary profile-derived features and simulates the RNA-interacting state of every amino acid residue in a given sequence. The resulting 16-dimensional multi-class output neural network was optimized by an early-stopping algorithm and five-fold-out cross-validation. Results indicate that specific RNA-binding contacts can be predicted with accuracy levels similar to those achieved in DNA-binding proteins. We applied dinucleotide-specific contact predictions to identify full-length RNA sequence targets of proteins, with promising success.

In summary, the proposed method can be used in two ways: (1) to improve the performance of RNA-binding sites on proteins, as prediction scores will be modulated by the dinucleotide composition of target RNA sequences and one can look for prediction scores that correspond to the dinucleotide enriched in a known target and (2) predicted binding sites in proteins can be converted to a 16-dimensional dinucleotide score, which works like a pseudo dinucleotide composition itself and candidate target RNA sequences can thereby be scored by comparing observed dinucleotide compositions in candidate target sequences and thereby use them to predict targets of RNA-binding proteins.

## Methods

### Data set (PRNA160)

The dataset and annotations have been adopted from our previous work on predicting RNA-binding proteins from their electric moments [18]. The data preparation procedure is summarized here as follows.

All protein-RNA complexes in the Protein Data Bank (PDB) [19], which have an annotations in SCOR database were selected [20]. We eliminated redundancy using BLASTCLUST [21] by obtaining clusters at a 25% sequence identity and selecting the chain with the highest number of RNA contacts from each of the 160 clusters obtained at this threshold. A final list of 160 protein chains and their structural classes, was used to generate and evaluate the models, and is provided as a supplementary Table S1 (Additional file 1).

### Contact profiles

The contact profiles for the protein sequences were computed from protein-RNA complex structures in the compiled data set. We defined a contact when the distance between any atom in the protein and any atom of the RNA dinucleotide is less than 3.5Å. The contact class of all 16 dinucleotide sequence elements is written in alphabetically order viz. AA, AC, AG, AU, CA, CC, CG, CU, GA, GC, GG, GU, UA, UC, UG and UU.

### Contact statistics and random docking

The statistical significance of contact preferences was evaluated using a Chi-squared test with one degree of freedom. This requires a background contact frequency, which is computed using a random docking procedure, as has been previously used for DNA-binding proteins [16,22]. In this procedure, all observed contacts in each protein-RNA complex were divided into unique amino acid-dinucleotide pairs (20x16 combinations) in accordance with the products of accessible surface area (ASA) of the residue and the dinucleotide in question and the expected number of contacts (Eij) is given by:(1)

Where i, and j refer to 20 unique residues and dinucleotides and ASA refers to the absolute accessible surface area, computed by the NACCESS program [23]. Coordinates for each protein chain were isolated from the complex structure before computing the ASAs of amino acid residues. RNA-chains were isolated similarly for dinucleotide ASAs. ASA(i) refers to the total accessible surface area of all residues of a giventype in a given data set and N is the total number of observed contacts of all residue-dinucleotide pairs.

Both observed and expected numbers of contacts were computed for each complex and data were pooled to get the overall chi-squared values for each dinucleotide-amino acid pair as follows:(2)

Where i and j have the same meaning as in equation (1) i.e. they take values from 1 to 20 and 1 to 16 respectively. E_ij_ is computed as in (1) and O_ij_ is the corresponding number of contacts in all complexes (note that (1) is for one complex but (2) refers to all complexes taken together). Chi-squared values from observed and expected number of contacts are converted to p-values using standard look-up tables. Chi-squared values computed above denote statistical significance of both positive and negative deviations from expected numbers of counts. To indicate the exclusion or enrichment of a residue-dinucleotide pair in the interface, signed chi-squared values were obtained by multiplying these values by -1 if the observed number of contacts was less than the expected counts.

### Sequence features

Two types of encoding schemes of single protein sequences are widely prevalent and have been adopted for this work. A first scheme represented an amino acid residue and its neighbours by 21-bit-sparse-encoded binary vectors i.e. by taking all the 21 components to be zero except for the one identifying a given residue, which is set as 1. The first 20 components resemble the amino acid type and the last one is labelling terminal positions, without neighbours. A second scheme represented each residue by its evolutionary profile. As previously described by us and others [24,25], the evolutionary profile of a residue, represented as Position-Specific Scoring Matrices (PSSMs), was computed by using the PSI-BLAST program and against NCBI's NR database for each protein sequence. PSSMs were generated by three iterations of PSI-BLAST with default parameters of *blastpgp*. Prior to neural network training, a vector representing global amino acid composition (GAC) of each protein sequence was concatenated to the feature vector of each residue. Sliding windows of sizes ranging from 1 to 8 sequence neighbours were centred on each residue. Different combinations of feature matrices were tested and the best performing combination was retained.

### Artificial neural networks

Three layered (one hidden layer) fully connected neural networks trained by back propagation were simulated using the SNNS software package [26]. The number of input nodes was equal to the number of training features and 16 output nodes represented all possible 16 RNA dinucleotide contacts. Different network architectures were optimized for every feature matrix varying the number of nodes in the hidden layer. Optimum networks were chosen by a five-fold cross-validation scheme according to an early stopping criterion. The data was divided into five sets of proteins and for each training cycle, three subsets were combined to form the training data. Out of the two subsets left out, one was used to determine the stopping point of training. All the reported performance scores were computed on the third (left out) sets. Shuffling the training and test data sets during five cycles of cross-validation ensured that each protein had been used for evaluating performance. Numerical values between 0 and 1 returned by the neural network were transformed into a binary state of binding or non-binding by varying cut-off values during receiver operating characteristic (ROC) analysis. For the purpose of constructing a web server, a precision score (for each of the 16 contact classes) was computed for a prediction score at a given position used as a cut-off. This score corresponds to the true positive probability of that contact class.

### Applicability to scanning interacting protein-RNA fragments

From the protein-RNA complexes in the PRNA160 dataset, pairs of protein and RNA sequence fragments of fixed lengths were generated by using a sliding window on both. Subsequently, all combinations of protein-RNA fragment pairs in a given complex were generated by matching each protein fragment to all RNA fragments. Fragment pairs were assigned a binary class according to the contacting information from each complex structure (0 or 1). A fragment pair was labelled as 1 if any of the atoms from the protein fragment was in contact with any of the atoms of the RNA fragment at a threshold of 3.5Å atom-atom distance; otherwise it was labeled as 0. A second neural network model was built to identify the positive class (label=1) from the negative class (label=0). The protein fragments were encoded in fragment feature vectors built by concatenation of the predicted 16 dinucleotide scores of each residue in a given fragment, while the RNA sequence fragments were encoded using dinucleotide compositions. The protein and RNA feature vectors for each fragment pair in a given complex were concatenated forming a fragment-pair feature matrix per complex that was used to train a new neural network. We used the same training algorithm described above but in this case the neural networks predict a one-dimensional binary vector encoding the “paired” or “non-paired” state of the protein-RNA fragment pairs.

### Performance evaluation

Neural networks are trained to return a vector of 16 dinucleotide-binding prediction scores for a protein residue between 0 and 1, which are transformed to binary states of binding or non-binding by choosing a cut-off (for the fragment-pair prediction, only one score is obtained instead of 16, and is treated in an obviously similar way). For each dinucletide type, the occurrence of a contact is considered positive (P) and negative (N) otherwise. True (T) indicates that the states of predicted and observed contact are identical and false (F) otherwise. The notation TP, TN, FP, FN combines these labels to return the number of data points (residues) in that category. These values correspond to a cut-off at which the neural network outputs are transformed into binary predictions. The predicted binding scores are transformed into binary predictions by using different cut-offs yielding sensitivity and specificity over the entire score range. ROC graphs for "1-specificity" (false positive rate) values versus corresponding sensitivity (true positive rate) values are plotted. This graph depicts the variations of the increment in false positive rate with an increase in the true positive rate. Throughout this work, the performance of the models was measured by the total AUC for the ROC.

## Results and discussion

### Dinucleotide contact statistics

A simple look at the number of contacts with RNA revealed that on an average 1.7% of the residues in the RNA-binding proteins are in contact with a given dinucleotide in the complexes (data not shown). This result differs from our previous report on DNA-binding proteins, which bind to DNA with an average of 1% of the residues, reflecting a larger contact surface in protein-RNA complexes. In contrast to the protein-DNA complexes, in which residues have remarkable preferences for particular dinucleotides such as AG/CT, AC/GT and AT/AT, the contacts in protein-RNA complexes are more homogeneously distributed among all dinucleotides (detailed data not shown). However, contacts with CG (2.2%), GA (2.2%), GG (2.2%), CC (2.1%) and AG (2.0%) were slightly more abundant than dinucleotides UA (1.5%), AU (1.2%) and UU (1.0%). This result illustrates the greater complexity of RNA recognition in comparison to DNA. This is expected, because Protein-RNA interactions involve the matching of a variety of conformational arrangements rather than recognition of canonical helix configurations, as in the case of protein-DNA complexes [27].

**Figure 1:**
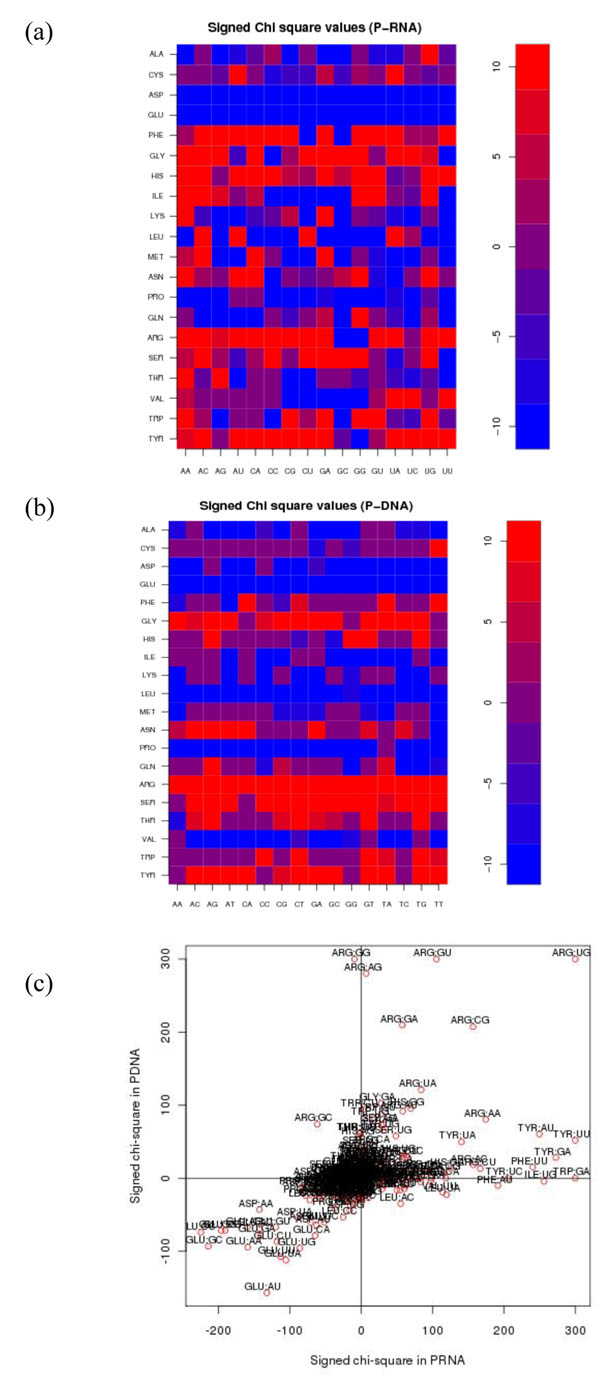
Chi-squared values of amino-acid dinucleotide contacts (a negative sign means the observed number was less than the expected value in that contact class). (a) Dinucleotide (x-axis) contact preferences with individual amino-acid residues (y-axis) in protein-RNA-complexes are displayed (high positive score means contacts are preferred). (b) Same as (a) but protein-DNA complex preferences are shown instead. (c) A scatter plot of contact preferences in protein-RNA versus protein-DNA complexes.

The binding preferences of the 20 amino acids towards the 16 dinucleotides and their comparison with DNA-binding proteins are shown in Figure 1 (see Table S2 in Additional file 2 for details). Similar to DNA-binding proteins (Figure 1(b)), amino acids Glu and Asp are excluded from the interface as expected. On the other hand positively charged residues i.e. Arg, Lys and His are enriched for most dinucleotide contacts with few exceptions. Interestingly, Phe and aromatic amino acids Trp and Tyr showed a significant enrichment for most dinucleotides. With regard to the specificity, we observe clear preferences for several cases. For example Lys has high positive scores only for purine dinucleotides AA and GA and low or statistically insignificant preferences for other nucleotides. On the other hand Arg has high scores for most but not for GC and GG dinucleotides. Figure 1(c) reveals the similarity and differences between RBPs and DBPs. As expected, most of the data are observed in the first and third quadrant, implying that the same residue-dinucleotide pairs are enriched in DBPs and RBPs. However, there are some exceptions. First, some pairs like Arg-AG, Arg-CG, and Arg-GA are enriched in DBPs but not in RBPs. On the other hand RBPs employ a larger set of residues as many hydrophobic residues pair with dinucleotides (e.g. Tyr-UU, Trp-GA, Tyr-AU and Phe-AU) whereas their pairing with corresponding DNA dinucleotides is much less significant. Thus, we conclude that a larger set of residues is employed for interactions in RBPs compared to DBPs and hydrophobic interactions are particularly important for conferring specificity in RBPs. This can be explained in view of the fact that nucleotide side chains (base atoms) are more exposed in RBPs due to single-stranded nature and hence more stacking interactions can occur than in DNA, where most interaction must occur with the phosphate backbone and hence interactions are more electrostatic in nature. The consistency of these preferences and the role of neighbours can also be revealed by estimating the prediction performance of models trained using this information.

### Prediction performance using sequence neighbours

**Figure 2:**
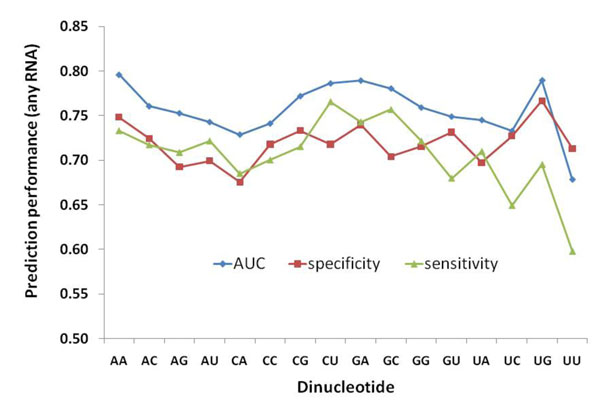
Performance of predicting contacts with 16 unique dinucleotides. Area under the ROC curve, specificity and sensitivity at peak F-score are plotted for the cross-validated models in terms of their ability to predict protein-RNA contacts corresponding to each of the possible 16 specific dinucleotides.

**Figure 3:**
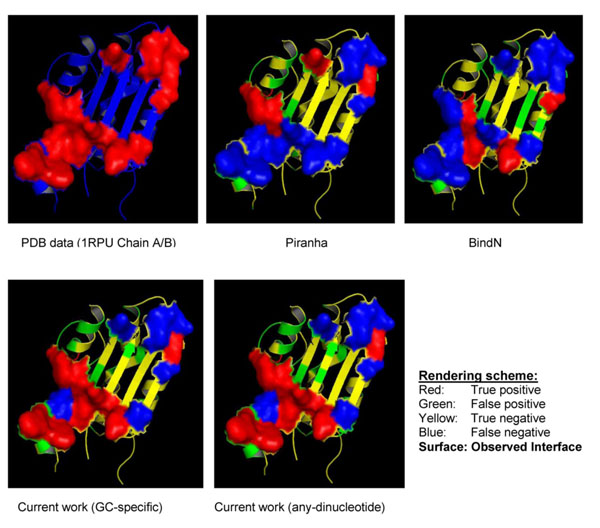
Comparison of prediction results of traditional non-specific RNA-binding site prediction approaches [12,13] with the proposed method. Figure shows that incorporating dinucleotide information improves resolution of RNA-binding surface and reduced the false positive rate.

Sequence and evolutionary information was used to model the dinucleotide contacting states of all protein residues in the PRNA160 data. Neural networks were trained with different feature matrices combining 21-bit sparse-encoded binary vectors, PSSM scores at differently sized neighbour windows and GAC scores of the whole protein sequence. Neural networks yielded an optimum model with 10 hidden nodes trained with a feature matrix of 320 input vectors. The combination of evolutionary information of the predicted residue and two neighbours with GAC values of the whole sequence yielded the optimum feature matrix. Figure 2 depicts ROC graphs of the “best” neural network with AUC scores of ~67-79%. The lowest accuracies <70% correspond to contacts with UU dinucleotides, whereas the contacts with AA, CU, GA and UG dinucleotides were predicted with the highest accuracies of about 80%. Notably, the optimum contribution of sequence neighbours to the dinucleotide-specific binding extends only up to 2 residues, representing a 5-residue fragment in the protein sequence. No further improvement in performance could be obtained by increasing the window size beyond 5 residues, apparently because the distant neighbour information is too complex to be captured by the amount of data we have. Performance levels are generally lower than but remain comparable to the reported accuracies for general RNA-binding site prediction from sequence, which are in the range of 80-85% [8-11], highlighting the difficulties in the prediction of specific contacts in comparison to general RNA-contact prediction. Yet, the results are encouraging as they are likely to improve in the future with more abundant data sets. In particular, the proposed method is likely to enable us to identify binding sites more accurately when provided with known target RNA-sequences. Ability of dinucleotide-contact prediction to fine-tune binding site prediction on RBPs in reference to the dinucleotide sequence on the RNA-target is demonstrated in Figure 3. Using two publicly available web servers in [11,13], we show that our proposed approach can enhance identification of interacting regions. There are not many examples forming contacts with only one dinucleotide type, which makes the visual or statistical presentation of this result more difficult, but the illustrated example is quite informative. The overall ability of the proposed model to identify specific contacts is obvious, as general binding site algorithms cannot distinguish between RNA-targets at all and any progress in that direction is a clear advantage over the general approach.

### What leads to the predictability of specific contacts from PSSMs?

PSSMs, as used in this work enumerate the residue substitution profiles during evolution at each alignment position for a given protein sequence. The evolutionary patterns of amino acid substitutions at given positions of RBPs represent constrains imposed by the requirement of specific interactions. In our previous study on dinucleotide-specific DNA binding-site prediction [16], we also found that the amino acid substitution pattern of a residue and its neighbours (encoded in the PSSM) was well-defined during evolution and could be correlated to the ability of the residue to interact with particular DNA dinucleotides. Our findings for protein-RNA interactions suggest that, although more complex and varied than protein-DNA interactions, the substitution patterns of the functional residues in RNA-binding proteins are also significantly constrained during evolution.

### Predictability of specific contact across RNA functional classes

**Figure 4:**
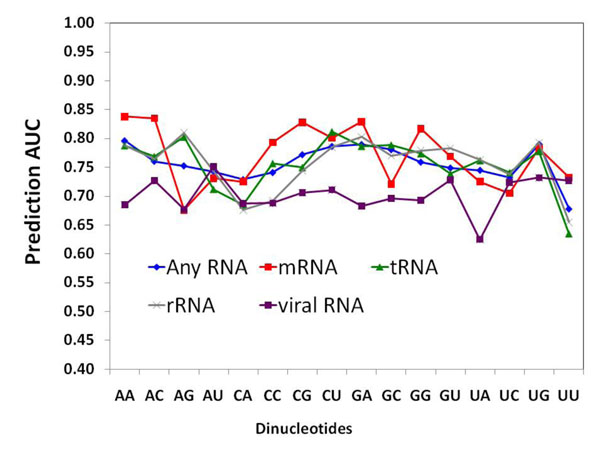
Prediction performance for various RNA-binding protein classes. Protein-RNA complexes were grouped by their functional class and the prediction performance of our models within each category were evaluated.

Besides playing a broad range of roles in the cell, RNA molecules might also mediate unknown biological functions [28,29]. Two main RNA recognition classes have been defined: groove binding, in which a protein secondary structure element is positioned into the groove of an RNA helix; and beta-sheet binding, in which beta-sheet surfaces create pockets to bind unpaired RNA bases [30]. The diversity of RNA-binding patterns has been previously discussed in the context of functional groups in which RNA binds different protein secondary elements [22]. However, it is not straightforward to separate groove-binding proteins from beta-sheet binding ones, as the two modes of binding often occur together in complexes. It is much more convenient to classify RNA-binding proteins in terms of the target RNA function. Specifically, we evaluated the performance of the optimum neural network according to the functional class of the complexes. We considered four functional classes of RNA: viral RNA, mRNA (messenger RNA), tRNA (transfer RNA) and rRNA (ribosomal RNA) representing about 9.9%, 15.1%, 31.1%, 32.1% of all contacts in the dataset, respectively. Figure 4 depicts the prediction performance of our model for proteins binding to different RNA classes. We find that the optimum neural network performed quite homogeneously for all RNA functional classes except the viral complexes (Figure 4) which resulted in low accuracies in the AUC range of 62-77% in comparison to the accuracies for mRNA, tRNA and rRNA complexes, which range between 68-84%, 64-80% and 66-81%, respectively. These results suggest that viral RNA-protein interaction patterns are different from the rest of the complexes. In fact, besides having the most polar and least well packed RNA binding sites among all RNA-binding proteins [31], viral protein-RNA complexes have been also reported to be the least sequence-specific [31], which could explain their poor performance on specificity prediction.

In order to improve the model performance for the different RNA functional classes, we implemented functional class-wise predictors of dinucleotide-specific RNA binding sites in proteins. In this case, neural networks were trained independently with complexes from a single functional class. After trying all combinations of sequence and evolutionary features for each RNA class, the prediction performance only partially improved for proteins binding to viral RNA. Interestingly in this case, the optimum neural network was trained with evolutionary information of the interacting residue and GAC of the whole protein sequence without considering any contribution from neighbouring residues. Despite the low accuracy (AUC<55%) yielded for AU dinucleotide (Figure S1 in Additional file 3), the AUC values for the rest of the dinucleotides ranged from 63-88%. It is noteworthy that CU, GA, and UG dinucleotides exhibited improved accuracies of about 85%, 81% and 88%. The data shortage on viral protein-RNA crystal structures is likely another cause of the underperformance observed for this RNA functional class, which could be overcome when more crystal structures of viral RNA complexes become available.

### Application to the prediction of binding protein-RNA fragment pairs

**Figure 5:**
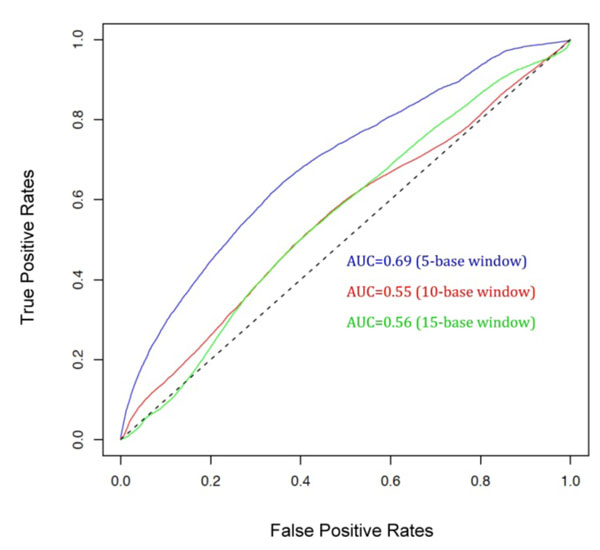
Performance of model trained to predict RNA targets of RBPs. Using various RNA-sequences, dinucleotide contact prediction scores from proteins were transferred to each position on the RNA sequence, based on dinucleotide composition and corresponding peak prediction score. The ability of the model to score RNA sequences better in correspondence to correct protein partners was evaluated in contrast to high scoring RNA sequences in reference to wrong partners.

A second neural network model was implemented to evaluate the ability of the dinucleotide specific RNA-binding scores to discriminate between “binding” and “non-binding” protein-RNA fragments in a given complex. By shifting one residue at a time, protein and RNA sequences were scanned to obtain all protein and RNA fragments of a given length per complex. All possible combinations of protein-RNA fragment pairs were generated for a given complex. Fragment pairs were labeled as “binding” if the fragment pair contains binding sites and “non-binding” otherwise. Neural networks were trained with fragment pair features obtained by concatenation of the predicted 16 dinucleotide-binding scores of each residue in a protein fragment and the dinucleotide composition of the corresponding RNA fragment of the pair. Figure 5 shows ROC plots and AUC values for the optimum models to predict protein and RNA fragments binding at different lengths. The accuracy to recognize binding fragment pairs varied with the fragment lengths. Interestingly, binding fragment pairs of five residues, which represent the same window size of the optimum dinucleotide specific RNA-binding model, were recognized with the highest accuracy of about 70%. According to the recognition accuracy of RNA fragments binding to specific protein sequence fragments, our methodology seems very promising for the discovery of putative RNA functional elements on a genomic scale.

### SRC PRED web server

A web server which takes FASTA-formatted sequences as input and predicts 16-dimensional vectors representing all possible dinucleotides for each residue position has been implemented and made available at http://tardis.nibio.go.jp/netasa/srcpred. One of the problems in working with this type of web servers is to interpret prediction scores. In this regard, we developed a strategy by using the raw prediction score at each position as a cutoff and determined the corresponding precision in the benchmarking data sets. Precision scores at this cutoff represents the probability that the position being in the positive class i.e. binding residue-dinucleotide pair. The web server automatically returns these probability scores for all residue positions and provides a graphical prediction highlighted according to the range of these scores.

## Conclusions

The paper shows that single amino-acid sequences and evolutionary profiles can predict RNA dinucleotide-specific contacts with accuracy somewhat lower than the prediction of general RNA-binding sites. Specific contacts in different RNA functional classes can be successfully predicted from a single comprehensive model but viral RNA complexes performance was slightly poorer. The best prediction accuracies, measured by the area under the ROC graphs, were ~80% for the general model. In addition, we showed that the calculated residue-wise prediction scores, used in combination with dinucleotide compositions correctly identified about 70% of protein-RNA fragment pairs at complex interfaces. This study will provide us with a better understanding and more accurate predictions of the specific base-amino acid interactions in protein-RNA complexes.

## Authors' contributions

SA conceived of and designed the study. MF implemented it in consultation with DS, YK, AS and KM. SA developed the web server. Manuscript was prepared by SA and MF on which AS, KM, DMS, and YK gave useful suggestions and improved it. All authors read and approved of the manuscript.

## Competing interests

The authors declare that they have no competing interests.

## Supplementary Material

Additional file 1 **Various RNA-binding proteins.** Zipped file, which contains lists of various RNA-binding proteins in text format (Table S1). Click here for file

Additional file 2 **Expected and observed contacts and chi- squared statistics.** Detailed values of expected and observed of contacts and chi- squared statistics (Table S2). Click here for file

Additional file 3 **ROC plots of prediction performance per each dinucleotide class.** ROC plots of prediction performance per each dinucleotide class (Figure S1). Click here for file
