# Supplementary material for: Prediction of dinucleotide-specific RNA-binding sites in proteins
Source: BMC Bioinformatics. 2011 Nov 30;12(Suppl 13):S5. doi: 10.1186/1471-2105-12-S13-S5 (PMC3278845; doi:10.1186/1471-2105-12-S13-S5)
Supplement: Additional file 3 — ROC plots of prediction performance per each dinucleotide class. ROC plots of prediction performance per each dinucleotide class (Figure S1). [file 1471-2105-12-S13-S5-S3.pdf]

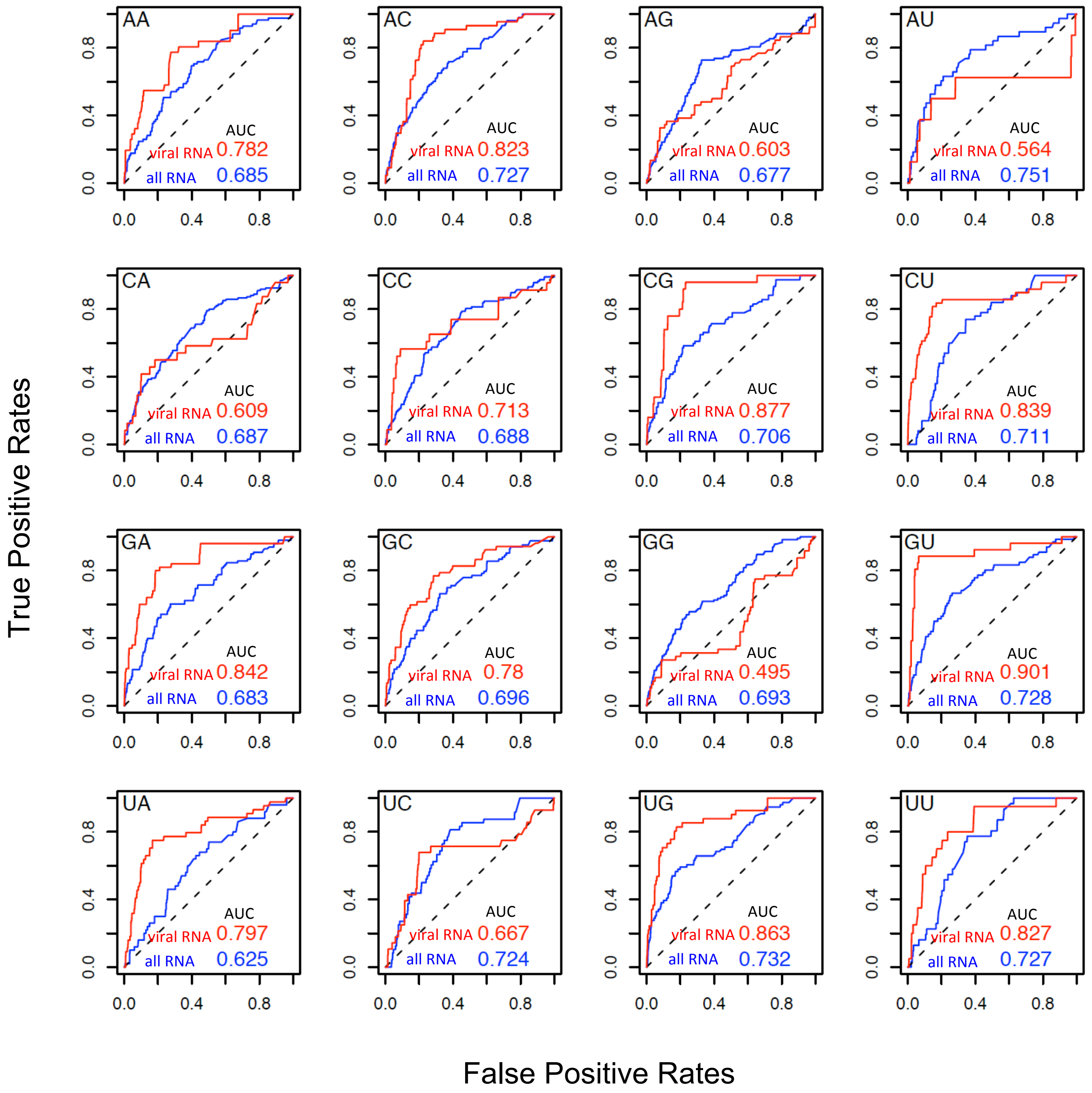

Figure. ROC curves and AUC values for the prediction of the 16 RNA dinucleotides contacts for viral RNA using the optimum neural network trained with all RNA complexes (blue) and a neural network trained only with viral RNA complexes (red).
